# Dose–Response Relationship between Environmental Exposure to Nickel and Pulmonary Function in the Korean General Population Aged 40 or Older

**DOI:** 10.3390/ijerph18137016

**Published:** 2021-06-30

**Authors:** Joon-Sung Joh, Mo-Yeol Kang, Jun-Pyo Myong

**Affiliations:** 1 Department of Internal Medicine, National Medical Center, Seoul 04564, Korea; ssabana777@gmail.com; 2 Department of Occupational and Environmental Medicine, Seoul St. Mary’s Hospital, College of Medicine, the Catholic University of Korea, Seoul 06591, Korea; snaptoon@naver.com

**Keywords:** nickel, pulmonary function, environment, exposure, dose–response relationship

## Abstract

Nickel is a well-known skin allergen; however, few studies to date have investigated the association between nickel exposure and lung function impairment. The present study, therefore, evaluated the relationship between blood nickel concentrations and lung function profiles in the Korean general population (*n* = 1098). Dose–response relationships between blood nickel quartiles and pulmonary function were assessed by sex in multivariate models, after adjustment for potentially confounding factors such as age, height, and smoking status. Quartiles of blood nickel concentrations were significantly associated with markers of pulmonary function in Korean men, such as forced expiratory volume in 1 second (FEV_1_) and forced expiratory flow 25–75% (FEF_25–75%_). Relative to the first quartile, the estimated coefficients (standard error (SE)) of blood nickel levels for FEV_1_ in the third and fourth quartiles of Korean men were −126.6 mL (59.1) and −138.5 mL (59.8), respectively (*p* < 0.05). Relative to the first quartile, the estimated coefficients (SE) of blood nickel levels for FEF_25–75%_ in the second and fourth quartiles were −244.9 mL (109.5) and −266.8 mL (111.5), respectively (*p* < 0.05). Dose–response relationships were observed between quartiles of blood nickel concentrations and the pulmonary function markers FEV_1_ and FEF_25–75%_ in Korean men aged 40 or older.

## 1. Introduction

Nickel is a ubiquitous material in the environment, being present in soil, grains, and water. Owing to its high ductility and low levels of abrasion and corrosion, nickel alloys have been widely used for industrial purposes, such as in stainless steel [1]. Humans are exposed to and absorb environmental nickel through the respiratory system, food intake, and skin exposure [2,3,4].

Inhaled nickel shows diverse respiratory manifestations from local inflammation, clinical disorders, and lung cancer. Chronic nickel inhalation is associated with alveoli inflammation and reactive oxidative stress. Chronic oxidative stress may cause lung cancer among nickel-exposed workers. Airway disorders related to nickel exposure also have been reported [5,6,7,8,9]. Although nickel can act as an allergen [5], few large-scale epidemiological studies, to date, have assessed the association between nickel exposure and airway diseases [6,7,8]. In addition, a population-based study showed a statistically significant association between blood nickel concentration and respiratory disorders; however, the association was not strong [9]. Because the blood concentration of nickel is low in the general population, clinically apparent diseases, such as asthma or other respiratory diseases, might be not a proper outcome to assess exposure to nickel. Therefore, it is necessary to find a proper health outcome, such as pre-clinical parameters, to evaluate the effects of exposure to nickel exposure in the general population. 

The present study, therefore, investigated the relationship between blood nickel concentration and lung function profiles in members of the Korean general population enrolled in the 2017 Korean National Health and Nutrition Examination Survey (KNHANES).

## 2. Materials and Methods

### 2.1. Study Materials and Population

The KNHANES is a nationwide database of non-institutionalized individuals representative of the general population of South Korea, using stratified, multistage, and clustered methods. Details of KNHANES have been described elsewhere [10]. Because nickel concentrations were measured only during the Seventh KNHANES in 2017, data from this survey were used in the present study. Spirometry data were obtained for those aged 40 or older. Of the 8127 participants in KNHANES 2017, those lacking information about spirometry (*n* = 4529), those without heavy metal profiles (*n* = 2499), and one patient lacking imputation of nickel concentration (*n* = 1) were excluded in this study. The present study, therefore, included 1,098 members of the Korean general population aged 40 or older.

### 2.2. Measurement of Blood Nickel Concentration

Blood nickel concentrations were measured by Green Cross Laboratory (GC Labs), which also performed background quality control. Briefly, 5 mL of blood was extracted with EDTA by a trained medical technician, and the containers were delivered to GC Labs under the KCDC KNHANES quality control protocol. Blood nickel concentrations were measured with an inductively coupled plasma mass spectrometer (ICP-MS) system (7900; Agilent, Tokyo, Japan) [11], with a lower limit of detection of 0.1 µg/L. For internal quality control, nickel concentrations were measured in the Blood Metals Control Level 1,2 with Trace elements W.B control−1 (Seronorm, SERO, Billingstad, Norway), with a coefficient of variation (CoV) of 3.495–5.973%, and the German External Quality Assessment Scheme (G-EQUAS) 58-1A (Friedrick Alexander University, Erlangen, Germany), with a CoV of 1.321–3.523%. For external quality control, nickel concentrations were measured by G-EQUAS and the Quebec Multielement External Quality Assessment Scheme (QMEQAS, The Centre de toxicologie du Québec, Québec, Canada). 

### 2.3. Spirometry 

Spirometry was performed for those aged 40 or older in the 2017 KNHANES. Forced vital capacity (FVC), forced expiratory volume in 1 second (FEV_1_), FEV_1_/FVC, and forced expiratory flow 25–75% (FEF_25–75%_) were evaluated in participants. Explanation and meaning of the spirometry were described previously [12]. For quality control, spirometry was calibrated using a digital computed spirometer (Vyntus Spiro, CareFusion, San Diego, CA, USA). The details of quality control, such as calibrating methods, were described elsewhere [13]. 

### 2.4. Other Factors

Participant height was measured using a digital mobile stadiometer (Seca 274, Seca, Hamburg, German). Age was classified into four groups (40–49, 50–59, 60–69, and ≥70 years). Smoking status was determined using a structured questionnaire administered by a specially trained interviewer. Participants who answered “yes” to the question “Are you smoking currently?” were considered current smokers. Those who answered “no”, but had smoked at least 100 cigarettes during their entire lives, were regarded as ex-smoker, whereas those who had smoked <100 cigarettes were classified as never-smokers.

### 2.5. Statistical Analysis

Age and height were reported as arithmetic means and standard deviations (SD). Because the distribution of blood nickel concentration was skewed, it was log-transformed and described as the geometric mean (GM) and geometric standard deviation (GSD). The generalized additive model (GAM) was used to plot the distributions of pulmonary function profiles and log-transformed blood nickel concentrations with estimated values of the fitted smoothing splines of means and their 95% confidence intervals (95% CIs). Because GAM methods show a non-parametric association between log-transformed blood nickel concentrations and pulmonary function profiles, blood nickel concentrations were classified into quartiles by sex. The first through fourth quartiles were Ni < 0.235 µg/L, 0.235 µg/L< Ni ≤0.289 µg/L, 0.289 µg/L < Ni ≤0.367 µg/L, and 0.367 µg/L ≤Ni, respectively, for men; and Ni < 0.258 µg/L, 0.258 µg/L< Ni ≤0.316 µg/L, 0.316 µg/L< Ni ≤0.379 µg/L, and 0.379 µg/L ≤Ni, respectively, for women. The *p* for trend test was performed to estimate the trend of pulmonary profiles in each sex by blood nickel quartile. A univariate generalized linear model was utilized to estimate the coefficients and standard error (SE) of age category, height, smoking status, and blood nickel quartile levels for pulmonary function profiles (FVC, FEV_1_, FEV_1_/FVC, and FEF_25–75%_). Multivariate generalized linear analysis was performed after adjustment of the essential covariates for estimating lung function (age group and height [14]; because the participants were of a single ethnicity, ethnicity was not included). Because of the potential correlation between age and metal concentration, potential interaction between age and metal concentration should be interpreted in the multivariate analysis. Therefore, an interaction analysis was also performed. An interaction term, age*log-transformed blood nickel level, was inserted to validate the effect of this interaction on pulmonary function in multivariate generalized models. The plot of fitted smoothing splines was determined using the ‘gamm4’ package (version 0.2–6) of R version 3.51 (R Foundation for Statistical Computing, Vienna, Austria) [15]. Other statistical analyses were performed using SAS version 9.4 (SAS Institute, Inc., Cary, NC, USA). 

### 2.6. Ethics Approval

The design of the present study was approved by the Institutional Review Board of Seoul St. Mary’s Hospital (ID: KC21ZESI0090). Because KNHANES data are publicly accessible, informed consent was waived. 

## 3. Results

The present study analyzed 1,098 participants; Table 1 shows their demographic and clinical characteristics, including the distribution of blood nickel concentrations relative to pulmonary function parameters, with stratification by sex. The 1,098 subjects included 504 men, of mean ± SD age 57.3 ± 10.9 years, and 594 women, of mean age 55.8 ± 10.0 years. GM (GSD) blood nickel concentrations were 0.310 (0.106) µg/L in men and 0.318 (0.096) µg/L in women. Of the men and women included in this study, 34.1% and 4.2%, respectively, were current smokers. 

**Figure 1:**
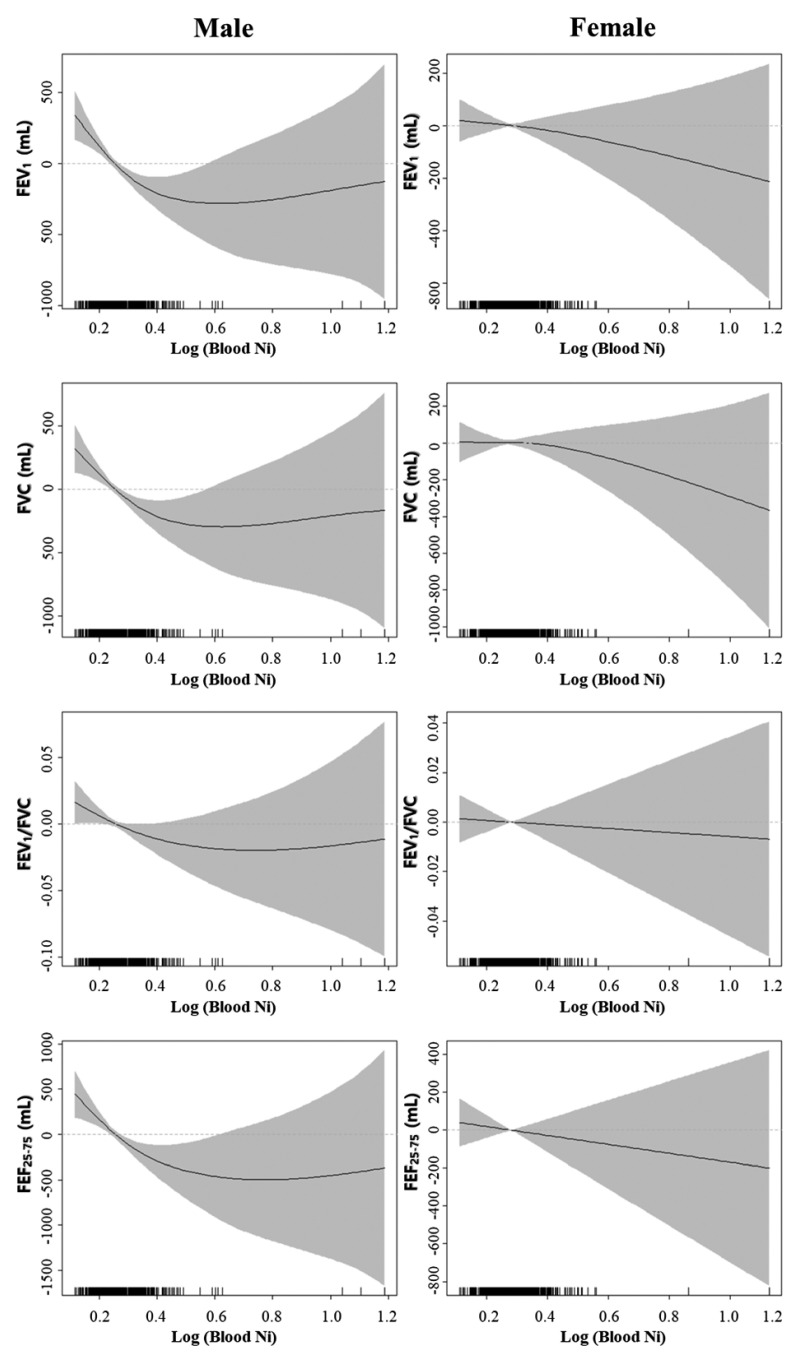
Smoothing plots with a general additive model showing the distribution of pulmonary function profiles by log-transformed blood nickel concentrations in men and women.

The general additive model in Figure 1 shows the distribution of pulmonary function profiles according to log-transformed blood nickel concentrations in men and women by sex. FVC, FEV_1_, FEV_1_/FVC, and FEF_25–75%_ correlated negatively with log-transformed blood nickel concentrations in Korean males aged 40 or older. However, these pulmonary parameters did not correlate with log-transformed nickel concentrations in Korean females aged 40 or older. 

Table 2 shows the pulmonary function profiles by sex and blood nickel levels. Parameters of lung capacity and function, such as FVC, FEV_1_, FEV_1_/FVC, and FEF_25–75%_, were significantly associated with quartiles of blood nickel concentrations in men. 

Univariate analyses of the estimated coefficients of independent variables for each lung function parameter are shown in Table 3. The estimated coefficients of age group, height, and blood nickel quartiles were significantly associated with lung function parameters in men, whereas smoking status was not significantly related to lung function profiles. 

The multivariate analysis in Table 4 showed that, relative to the first quartile, the estimated coefficients (SE) of blood nickel levels for FEV_1_ in the third and fourth quartiles of Korean men were −126.6 mL (59.1) and −138.5 mL (59.8), respectively (*p* < 0.05). Moreover, relative to the first quartile, the estimated coefficients of blood nickel levels for FEF_25–75%_ in the second and fourth quartiles of Korean men were −244.9 mL (109.5) and −266.8 mL (111.5), respectively (*p* < 0.05). The interaction between age and log-transformed nickel concentration, however, was not statistically significant (*p* > 0.05).

## 4. Discussion

The present study showed that FEV_1_ and FEF_25–75%_ in Korean men were associated with increased blood nickel concentrations. In addition, dose–response relationships were observed between blood nickel levels and these pulmonary function parameters (FEV_1_ and FEF_25–75%_).

Inhalation is one of the major routes of exposure to nickel. Most inhaled nickel is translocated into the gastrointestinal tract from the respiratory tract via a mucociliary clearance defense mechanism, involving expectoration and alveolar macrophages [16]. About 20–35% of inhaled nickel deposited in the lungs is absorbed into the blood [16], with less than 10% remaining in the lung tissue [17]. The half-life of nickel in blood ranges from 11 to 54 hours, depending on its chemical and/or physical characteristics [18], whereas its half time until urinary excretion is about 17–48 hours [19]. Therefore, blood nickel levels are a good surrogate marker of recent exposure to nickel via the respiratory tract. 

Inhaled nickel may influence pulmonary function via several possible mechanisms. First, inhaled nickel and nickel compounds may induce inflammatory responses in alveoli. For example, a study in animals showed an increase in neutrophils in the lungs after exposure to nickel for 2–12 days [20]. In an in vivo study with male mice, increased protein concentration in bronchoalveolar lavage fluid and inflammatory cytokine-related mRNA expression alteration were observed after inhalation of nickel nanoparticles [21]. In a series of workers suffering from occupational asthma after exposure to nickel and chromium fumes during electroplating, metallic salts were suggested to be associated with irritant-induced asthma [22]. For environmental exposure, the Columbia Center for Children’s Environmental Health birth cohort study showed that ambient nickel exposure was strongly associated with alveolar inflammation among children aged 9–11 [23]. Therefore, focal deposits of nickel and its compounds may influence airway inflammation, reducing FEV_1_ and FEF_25–75%_. Second, systemic nickel allergy syndrome presents as extra-cutaneous symptoms, including respiratory tract symptoms caused by the ingestion of nickel-containing beverages [24]. Avoidance of nickel-containing foods was found to reduce nasal and bronchial symptoms, suggesting that nickel exposure may be associated with respiratory symptoms and reduced lung function [25]. Third, nickel may induce a type 1 hypersensitivity reaction, which may reduce FEV_1_ and FEF_25–75__%_. It is less likely to be a good potential mechanism. Although nickel-induced allergic contact dermatitis has been associated with a type IV hypersensitivity reaction, several case reports have described specific IgE antibodies against nickel and nickel–albumin conjugates [7,26]. Few studies, however, have evaluated specific IgE antibodies, suggesting the need for further studies to elucidate the possible mechanisms by which nickel reduces lung function.

Tobacco smoking may potentially confound the relationship between blood nickel levels and lung function [27,28]. Therefore, we performed an additional analysis of non-current smokers to control for the effect of smoking (Supplementary Table S1). Relative to the first quartile, the estimation coefficients (SE) of blood nickel levels for FEV_1_ in the third and fourth quartiles of Korean men were −174.8 (75.9) mL and −161.5 (72.8) mL, respectively, and the estimation coefficient (SE) of blood nickel levels for FEF_25–75%_ in the fourth, relative to the first, quartile was −278.0 (136.9) mL. Therefore, the smoking status may not be a potentially confounding factor in the present study. 

Age is a major determinant for lung function and was found to correlate with blood nickel levels. To validate the effect of age on the relationship between blood nickel concentration and lung function, a multivariate analysis with an interaction term was assessed. No interaction was observed (Table 4), possibly because, unlike cadmium, which has a long half-life (7–16 years) [29], age-associated accumulation of nickel is less likely. Therefore, even after adjustment for age and other lung function-related factors (height and smoking), lung function (FEV_1_ and FEF_25–75%_) continued to be related to blood nickel concentration. 

In contrast to Korean men, blood nickel concentration was unrelated to lung function in Korean women. Metallothionein (MT) is a metal-binding protein that reduces nickel-associated oxidative stress [30]. Female hormones (progesterone and 17 beta-estradiol) are known to inhibit the expression of MT mRNA [31]; thus, they may make women more vulnerable to nickel toxicity. Most of the women in the present study, however, were aged ≥50 years and, therefore, may have been post-menopausal. Post-menopausal women were likely protected from nickel toxicity by MT. The eligible female population might be associated with reducing any possible relationship between blood nickel levels and lung function in the present study. The difference between men and women might have been due to differences in the route of nickel exposure. Men are more likely to be exposed to nickel at the workplace, the main source of respiratory nickel exposure. Women, however, may be more prone to dietary intake of foods rich in nickel compounds [2]. Inhaled nickel might be associated with pulmonary function impairment. These factors may have confounded any nickel-related reduction in lung function. A further evaluation should be performed to evaluate the potential health outcomes according to intake routes.

### Strengths and Limitations

The present study had several limitations. First, the source of blood nickel (i.e., inhalation or food intake) was not evaluated in KNHANES. Second, the study design was cross-sectional, preventing a determination of the cause-and-effect relationships between nickel exposure and decreased lung function.

This study also had several strengths. The multivariable analysis enabled the adjustment of possible independent variables (e.g., age, height, smoking status) for lung function. Second, this study showed a possible dose–response relationship as well as an association between blood nickel concentration and decreased lung function. Third, the decreased lung function measured was a pre-clinical outcome evaluated in a general population containing a relatively large number of subjects. 

## 5. Conclusions

Blood nickel concentration was associated with reduced lung function in a general population of Korean men. Environmental exposure to nickel may influence preclinical responses, such as decreased pulmonary function in men. These findings indicate the need to reduce environmental exposure to nickel in vulnerable populations, including men with a predisposition to respiratory diseases (asthma, chronic obstructive lung diseases, etc.) and the elderly. Well-designed studies are needed to evaluate the routes of exposure to nickel (i.e., respiratory, food intake, and other routes), as well as the ability of nickel to induce immunologic responses.

## Figures and Tables

**Table 1:** General characteristics and blood nickel concentrations in a Korean general population by sex.

		Male		Female		Total	
		Mean (SD) *n*/(%)	Blood Ni (µg/L) *	Mean (SD) *n*/(%)	Blood Ni (µg/L) *	Mean (SD) *n*/(%)	Blood Ni (µg/L) *
Age (year)		57.3 (10.9)		55.8 (10.0)		56.5 (10.4)	
	40–49	147 (29.2)	0.281 (0.074)	177 (29.8)	0.325 (0.080)	324 (29.5)	0.305 (0.079)
	50–59	160 (31.8)	0.300 (0.104)	226 (38.1)	0.314 (0.073)	386 (35.2)	0.308 (0.087)
	60–69	118 (23.4)	0.330(0.111)	127 (21.4)	0.325 (0.086)	245 (22.3)	0.327 (0.098)
	70≤	79 (15.6)	0.354(0.138)	64 (10.7)	0.363 (0.141)	143 (13.0)	0.358 (0.139)
Height (cm)		169.0 (6.3)		156.8 (6.0)		162.4 (8.6)	
Smoking status							
	Current smoker	172 (34.1)	0.291 (0.068)	25 (4.2)	0.316 (0.0806)	197 (17.9)	0.294 (0.070)
	Ex-smoker	245 (48.6)	0.328 (0.133)	32 (5.4)	0.303 (0.081)	277 (25.2)	0.325 (0.128)
	non-smoker	87 (17.3)	0.297 (0.075)	537 (90.4)	0.326 (0.088)	624 (56.9)	0.322 (0.087)
Blood nickel level ^†^							
	1st Quartile	126 (25.0)	0.198 (0.023)	147 (24.7)	0.213 (0.028)	273 (24.9)	0.205 (0.025)
	2nd Quartile	126 (25.0)	0.263 (0.012)	149 (25.1)	0.286 (0.013)	275 (25.1)	0.275 (0.013)
	3rd Quartile	125 (24.8)	0.322 (0.016)	149 (25.1)	0.345 (0.014)	274 (24.9)	0.336 (0.016)
	4th Quartile	127 (25.2)	0.469 (0.139)	149 (25.1)	0.465 (0.095)	276 (25.1)	0.468 (0.117)
Total		504 (45.9)	0.310 (0.106)	594 (54.1)	0.325 (0.088)	1,098 (100.0)	0.318 (0.096)

Ni: nickel; SD: standard deviation. *: described as geometric means (GM) and geometric standard deviation (GSD). ^†^ For males: 1Q (Ni < 0.235 µg/L), 2Q (0.235 µg/L < Ni ≤ 0.289 µg/L), 3Q (0.289 µg/L < Ni ≤ 0.367 µg/L), 4Q (0.367 µg/L ≤ Ni); for females: 1Q (Ni < 0.258 µg/L), 2Q (0.258 µg/L < Ni ≤ 0.316 µg/L), 3Q (0.316 µg/L < Ni ≤0.379 µg/L), 4Q (0.379 µg/L ≤ Ni).

**Table 2:** Pulmonary function profiles according to blood nickel level quartiles and sex.

Blood Nickel Level *		FVC (mL)			FEV_1_ (mL)			FEV_1_/FVC			FEF_25–75_ (mL)		
		Mean	SD	*p*-Value	Mean	SD	*p*-Value	Mean	SD	*p*-Value	Mean	SD	*p*-Value
Male													
	1st Quartile	4160.9	684.9	0.004	3192.9	603.3	<0.001	0.766	0.067	0.012	2901.5	1070.6	<0.001
	2nd Quartile	3998.7	745.9		2974.8	666.6		0.741	0.085		2502.5	1024.7	
	3rd Quartile	3882.3	759.9		2932.0	619.8		0.757	0.072		2559.0	1023.9	
	4th Quartile	3823.6	699.0		2826.6	699.0		0.735	0.085		2312.7	1077.6	
Female													
	1st Quartile	2832.2	447.9	0.925	2239.3	399.2	0.843	0.790	0.063	0.670	2258.1	778.2	0.729
	2nd Quartile	2872.8	512.0		2287.1	420.3		0.797	0.053		2324.2	731.1	
	3rd Quartile	2904.3	525.0		2305.0	455.4		0.792	0.051		2303.6	773.7	
	4th Quartile	2862.8	518.7		2249.4	426.1		0.787	0.062		2227.9	730.7	

SD: standard deviation; FVC: forced vital capacity; FEV_1_: forced expiratory volume in 1 second; FEF_25–75_: forced expiratory flow. * For males: 1Q (Ni < 0.235 µg/L), 2Q (0.235 µg/L < Ni ≤ 0.289 µg/L), 3Q (0.289 µg/L < Ni ≤ 0.367 µg/L), 4Q (0.367 µg/L ≤ Ni); for females: 1Q (Ni < 0.258 µg/L), 2Q (0.258 µg/L < Ni ≤ 0.316 µg/L), 3Q (0.316 µg/L < Ni ≤ 0.379 µg/L), 4Q (0.379 µg/L ≤ Ni).

**Table 3:** Univariate analysis of independent variables, including blood nickel concentration, affecting pulmonary function components by sex.

			FVC			FEV_1_			FEV_1_/FVC			FEF_25–75_		
			Estimate	SE	*p*-Value	Estimate	SE	*p*-Value	Estimate	SE	*p*-Value	Estimate	SE	*p*-Value
Male	Age	40–49	1246.6	88.6	<0.001	1213.7	73.0	<0.001	0.079	0.010	<0.001	1664.0	126.3	<0.001
		50–59	780.3	87.4	<0.001	773.5	72.0	<0.001	0.058	0.010	<0.001	1145.4	124.5	<0.001
		60–69	541.1	92.5	<0.001	511.7	76.2	<0.001	0.040	0.011	0.001	621.4	131.8	<0.001
		70≤	ref			ref			ref			ref		
	Height (cm)		72.9	4.3	<0.001	60.5	3.8	<0.001	0.002	0.001	0.006	61.7	7.1	<0.001
	Smoke	Current smoker	ref			ref			ref			ref		
		Ex-smoker	−195.0	74.7	0.009	−114.0	65.6	0.083	0.005	0.008	0.493	−55.1	106.0	0.604
		non-smoker	−206.7	98.7	0.037	−43.9	86.6	0.612	0.030	0.010	0.003	202.5	139.9	0.148
	Blood Nickel level	1st Quartile	ref		*	ref		*	ref		*	ref		*
		2nd Quartile	−162.2	93.9	0.085	−218.2	81.5	0.008	−0.025	0.010	0.009	−399.0	131.9	0.003
		3rd Quartile	−283.9	94.3	0.003	−257.0	81.9	0.002	−0.008	0.010	0.404	−328.4	132.4	0.013
		4th Quartile	−327.6	93.9	0.001	−357.1	81.5	<0.001	−0.031	0.010	0.002	−577.2	131.9	<0.001
Female	Age	40–49	922.6	61.0	<0.001	873.4	48.8	<0.001	0.055	0.008	<0.001	1148.7	97.0	<0.001
		50–59	671.1	59.2	<0.001	633.6	47.4	<0.001	0.044	0.008	<0.001	868.8	94.1	<0.001
		60–69	431.7	64.1	<0.001	402.9	51.3	<0.001	0.028	0.008	0.001	493.6	101.9	<0.001
		70≤	ref			ref			ref			ref		
	Height (cm)		53.5	2.7	<0.001	41.6	2.4	<0.001	0.000	0.000	0.642	33.5	5.0	<0.001
	Smoke	Current smoker	ref			ref			ref			ref		
		Ex-smoker	15.7	133.6	0.907	42.1	113.5	0.711	0.005	0.015	0.743	118.4	200.9	0.556
		non-smoker	−124.2	102.4	0.226	−86.2	87.0	0.322	0.004	0.012	0.713	−75.0	154.0	0.626
	Blood Nickel level	1st Quartile	ref			ref			ref			ref		
		2nd Quartile	40.6	58.4	0.487	47.8	49.5	0.335	0.007	0.007	0.314	66.1	87.6	0.451
		3rd Quartile	72.1	58.4	0.218	65.6	49.5	0.185	0.002	0.007	0.742	45.5	87.6	0.604
		4th Quartile	30.5	58.4	0.601	10.1	49.5	0.839	−0.004	0.007	0.588	−30.2	87.6	0.731

FVC: forced vital capacity; FEV_1_: forced expiratory volume in 1 second; FEF_25–75_: forced expiratory flow; SE: standard error. Blood nickel level for males: 1Q (Ni < 0.235 µg/L), 2Q (0.235 µg/L < Ni ≤ 0.289 µg/L), 3Q (0.289 µg/L < Ni ≤ 0.367 µg/L), 4Q (0.367 µg/L ≤ Ni); for females: 1Q (Ni < 0.258 µg/L), 2Q (0.258 µg/L < Ni ≤ 0.316 µg/L), 3Q (0.316 µg/L < Ni ≤ 0.379 µg/L), 4Q (0.379 µg/L ≤ Ni). *: *p* for trend test <0.05.

**Table 4:** Multivariate analysis of the effects of blood nickel quartiles on pulmonary function components by sex.

Blood Nickel Level	FVC			FEV1			FEV1/FVC			FEF25–75		
	Estimate	SE	*p* for Trend *p*-Value	Estimate	SE	*p* for Trend *p*-Value	Estimate	SE	*p* for Trend *p*-Value	Estimate	SE	*p* for Trend *p*-Value
Male			0.076			0.024			0.245			0.050
1st Quartile	ref			ref			ref			ref		
2nd Quartile	−55.2	70.0	0.430	−112.5	58.7	0.056	−0.019	0.009	0.038	−244.9	109.5	0.026
3rd Quartile	−150.4	70.4	0.033	−126.6	59.1	0.033	−0.001	0.009	0.992	−142.6	110.1	0.196
4th Quartile	−101.4	71.2	0.156	−138.5	59.8	0.021	−0.018	0.009	0.054	−266.8	111.5	0.017
Age☓Log-transformed Nickel *			0.723			0.550			0.338			0.698
Female			0.316			0.807			0.839			0.860
1st Quartile	ref			ref			Ref			ref		
2nd Quartile	19.4	41.8	0.643	36.7	35.3	0.298	0.009	0.006	0.161	78.5	77.8	0.314
3rd Quartile	55.8	41.8	0.183	64.7	35.2	0.067	0.006	0.006	0.311	83.6	77.8	0.283
4th Quartile	18.3	41.9	0.663	7.7	35.3	0.828	−0.001	0.006	0.946	−5.5	78.0	0.943
Age☓Log-transformed Nickel *			0.670			0.592			0.726			0.821

FVC: forced vital capacity; FEV_1_: forced expiratory volume in 1 second; FEF_25–75_: forced expiratory flow; SE: standard error. Adjusted by age group, smoking status, and height in the multivariate analysis. * Adjusted by age group, smoking status, height, age*log-transformed nickel level in the multivariate analysis. Blood nickel level for males: 1Q (Ni < 0.235 µg/L), 2Q (0.235 µg/L < Ni ≤ 0.289 µg/L), 3Q (0.289 µg/L < Ni ≤ 0.367 µg/L), 4Q (0.367 µg/L ≤ Ni); for females: 1Q (Ni < 0.258 µg/L), 2Q (0.258 µg/L < Ni ≤ 0.316 µg/L), 3Q (0.316 µg/L < Ni ≤ 0.379 µg/L), 4Q (0.379 µg/L ≤ Ni).

## Data Availability

Data available on request due to restrictions, privacy, or ethics. The data presented in this study are available on request from the corresponding author.

## Supplementary Materials

The following are available online at https://www.mdpi.com/article/10.3390/ijerph18137016/s1, Table S1. Multivariate analysis of the effects of blood nickel quartiles on pulmonary function components among non-current smokers by sex.
