# Supplementary material for: Dose–Response Relationship between Environmental Exposure to Nickel and Pulmonary Function in the Korean General Population Aged 40 or Older
Source: Int J Environ Res Public Health. 2021 Jun 30;18(13):7016. doi: 10.3390/ijerph18137016 (PMC8297347; doi:10.3390/ijerph18137016)
Supplement: Supplementary file 1 [file ijerph-18-07016-s001.zip › ijerph-1243320-supplementary.pdf]

Supplementary table legends

Supplementary table

Table S1. Multivariate analysis of the effects of blood nickel quartiles on pulmonary function components among non-current smokers by sex

Blood Nickel level	FVC			FEV1			FEV1/FVC			FEF25–75		
	Estimate	SE	p-value	Estimate	SE	p-value	Estimate	SE	p-value	Estimate	SE	p-value
**Male**												
**1st Quartile**	ref			ref			ref			ref		
**2nd Quartile**	−73.8	86.7	0.395	−106.1	73.9	0.152	−0.013	0.011	0.208	−193.9	138.8	0.163
**3rd Quartile**	−193.3	89.1	0.031	**−174.8**	**75.9**	**0.022**	−0.004	0.011	0.691	−254.3	142.7	0.076
**4th Quartile**	−132.0	85.5	0.108	**−161.5**	**72.8**	**0.027**	−0.014	0.010	0.178	**−278.0**	**136.9**	**0.043**
**Female**												
**1st Quartile**	ref			ref			ref			ref		
**2nd Quartile**	11.5	41.6	0.786	33.8	35.8	0.345	0.009	0.007	0.151	73.5	79.9	0.358
**3rd Quartile**	60.0	42.8	0.161	**74.3**	**36.0**	**0.040**	0.008	0.007	0.209	100.7	80.2	0.210
**4th Quartile**	9.5	42.8	0.826	4.7	36.0	0.894	0.002	0.007	0.978	−8.7	80.2	0.914

FVC: forced vital capacity; FEV_1:Forced expiratory volume in 1 second; $FEF_{25–75}$:forced expiratory flow; SE: Standard Error

Blood Nickel level: for males: 1Q: (Ni < 0.235 μg/L), 2Q: (0.235 μg/L< Ni ≤0.289 μg/L), 3Q: (0.289 μg/L<Ni ≤0.367 μg/L), 4Q: (0.367 μg/L ≤Ni); for females: 1Q: (Ni < 0.258 μg/L), 2Q: (0.258 μg/L< Ni ≤0.316 μg/L), 3Q: (0.316 μg/L< Ni ≤0.379 μg/L), 4Q: (0.379 μg/L ≤Ni)
